# Expression analysis of *Arabidopsis* XH/XS-domain proteins indicates overlapping and distinct functions for members of this gene family

**DOI:** 10.1093/jxb/ert480

**Published:** 2014-02-18

**Authors:** Haroon Butt, Sonja Graner, Christian Luschnig

**Affiliations:** Department of Applied Genetics and Cell Biology, University of Natural Resources and Life Sciences, Vienna (BOKU), Muthgasse 18, 1190 Wien, Austria

**Keywords:** *Arabidopsis*, gene family, protein localization, RdDM.

## Abstract

RNA-directed DNA methylation (RdDM) is essential for *de novo* DNA methylation in higher plants, and recent reports established novel elements of this silencing pathway in the model organism *Arabidopsis thaliana*. *I*NVOLVED IN *D*E *N*OVO DNA METHYLATION 2 (IDN2) and the closely related *F*ACTOR OF *D*NA *M*ETHYLATION (FDM) are members of a plant-specific family of dsRNA-binding proteins characterized by conserved XH/XS domains and implicated in the regulation of RdDM at chromatin targets. Genetic analyses have suggested redundant as well as non-overlapping activities for different members of the gene family. However, detailed insights into the function of XH/XS-domain proteins are still elusive. By the generation and analysis of higher-order mutant combinations affected in *IDN2* and further members of the gene family, we have provided additional evidence for their redundant activity. Distinct roles for members of the *XH/XS-*domain gene family were indicated by differences in their expression and subcellular localization. Fluorescent protein-tagged *FDM* genes were expressed either in nuclei or in the cytoplasm, suggestive of activities of XH/XS-domain proteins in association with chromatin as well as outside the nuclear compartment. In addition, we observed altered location of a functional FDM1–VENUS reporter from the nucleus into the cytoplasm under conditions when availability of further FDM proteins was limited. This is suggestive of a mechanism by which redistribution of XH/XS-domain proteins could compensate for the loss of closely related proteins.

## Summary

Activity of *Arabidopsis* XH/XS-domain proteins has been implicated in late events of RdDM, in close association with chromatin. Functional expression analysis now indicates partially redundant activities in nuclei and cytoplasm.

## Introduction

Redundant activities exhibited by closely related but distinct genes is a widely observed phenomenon in higher plants (Nowak *et al.*, 1997; Gottlieb, 2003; Gasciolli *et al.*, 2005; Vaucheret, 2008). Duplication of individual genes or even entire genomes results in the establishment of gene families, members of which frequently exhibit overlapping activities. In the model plant *Arabidopsis thaliana*, more than 80% of its present genome appears to have arisen from such duplication events (Cannon *et al.*, 2004). Once duplicated, loci might sustain redundant activities over an extended period of time. This predicts mechanisms by which homologous loci escape from the fate of pseudogenization by quickly diverging in functionality either by the acquisition of new functions (neofunctionalization; Ohno, 1970) or by subdivision of ancestral functions (subfunctionalization; Lynch and Force, 2000), or a combination of both (Qian *et al.*, 2010).

Ongoing functional diversification is exemplified by XH/XS-domain proteins, which constitute a gene family in the *Arabidopsis* genome, implicated in exerting redundant and distinct functions in the regulation of RNA-directed DNA methylation (RdDM) (Ausin *et al.*, 2012; Xie *et al.*, 2012*a*, *b*; Zhang *et al.*, 2012). *SUPPRESSOR OF GENE SILENCING 3* (*SGS3*) and the rice gene *X1* represent founder members of this plant-specific group of genes, both characterized by the occurrence of a single conserved XS domain (rice gene *X* and *SGS3*; Bateman, 2002). In *Arabidopsis*, nine additional proteins have been predicted, which, apart from the XS domain, encode a closely related XH (rice gene *X* homology) domain, located in the C-terminal portion (Bateman, 2002). Furthermore, XH/XS-domain proteins have been predicted to contain a coiled-coil domain, and six of the nine proteins encode a zinc finger (zf)-XS cysteine/histidine signature in the N-terminal portion of the protein (Bateman, 2002; Qin *et al.*, 2009).

*SGS3* was identified as a loss-of-function mutant exhibiting increased susceptibility to viral infections (Mourrain *et al.*, 2000), and was found to act in the generation of *trans*-activating small interfering RNAs (siRNAs) (Peragine *et al.*, 2004; Elmayan *et al.*, 2009), as well as in post-transcriptional gene silencing-induced viral/bacterial resistance and salt tolerance (Borsani *et al.*, 2005; Katiyar-Agarwal *et al.*, 2006). More recently, *Arabidopsis* XH/XS-domain proteins were characterized as regulators of DNA methylation (Ausin *et al.*, 2009; Zheng *et al.*, 2010; Finke *et al.*, 2012). Initially, a loss-of-function mutation in *INVOLVED IN DE NOVO DNA METHYLATION2* (*IDN2*)/*RNA-DIRECTED DNA METHYLATION12* (*RDM12*) was found to interfere with RdDM (Ausin *et al.*, 2009), whereas in another study *IDN2/RDM12* was identified by screening for release of silencing of a hypermethylated *RD29A::LUC* transgene (Zheng *et al.*, 2010). Apart from reduced DNA methylation in the promoter region of transgenic loci, endogenous loci were also affected in *idn2/rdm12* alleles, supporting the notion that IDN2/RDM12 acts in the RdDM pathway (Ausin *et al.*, 2009; Zheng *et al.*, 2010).

*In vitro* binding assays have demonstrated that IDN2/RDM12 and a close relative, termed *F*ACTOR OF *D*NA *M*ETHYLATION1 (FDM1; also described as *IDN2-LIKE1* and *IDN2 PARALOG1*), have the ability to bind dsRNA with a 5′ overhang (Ausin *et al.*, 2009; Xie *et al.*, 2012*a*). Moreover, a systematic analysis performed with *idn2* and mutants deficient in *FDM* paralogues demonstrated significant alterations in gene expression and DNA methylation that resembled further RdDM mutants (Xie *et al.*, 2012*a*). It was suggested that IDN2/RDM12 might act in an early step of siRNA generation, potentially facilitating their biogenesis (Ausin *et al.*, 2009; Zheng *et al.*, 2010; Xie *et al.*, 2012*a*). Alternatively, IDN2/RDM12 and related proteins were suggested to function in a later step of RdDM. This is supported by findings demonstrating that, whilst DNA methylation at RdDM target loci is affected in *idn2* alleles, generation of siRNAs is not, indicating that IDN2 is dispensable for this latter process (Ausin *et al.*, 2012; Finke *et al.*, 2012; Xie *et al.*, 2012*b*; Zhang *et al.*, 2012).

Analysis of RdDM target loci in *idn2/fdm* mutant combinations has indicated partially redundant activities within this gene family (Ausin *et al.*, 2012; Xie *et al.*, 2012*a*). In addition, studies performed by three laboratories demonstrated the formation of heteromeric protein complexes, consisting of IDN2 and two of its paralogues, with different subunits potentially exerting non-overlapping activities (Ausin *et al.*, 2012; Xie *et al.*, 2012*b*; Zhang *et al.*, 2012). The individual function of distinct XH/XS-domain proteins within such complexes, however, remains to be determined (Ausin *et al.*, 2012; Xie *et al.*, 2012*b*; Zhang *et al.*, 2012).

In this report, we have extended the analysis of *Arabidopsis* XH/XS-domain proteins and provide evidence for redundant activities by analysis of higher-order loss-of-function mutants. In addition, we performed expression and localization studies with *FDM* genes that indicated functional diversification within the XH/XS-domain family. We showed a differing subcellular distribution of closely related FDM proteins and present indications for intracellular protein relocation under conditions when the availability of additional FDM proteins is limited. This offers an explanation for the partially overlapping activities of XH/XS-domain proteins and highlights mechanisms by which members of this gene family could compensate for the loss of closely related proteins.

## Material and methods

### Plant growth and lines

Plants were grown on plant nutrient agar plates [5mM KNO_3_, 2mM MgSO_4_, 2mM Ca(NO_3_)_2_, 250mM KPO_4_, 70 μM H_3_BO_3_, 14 μM MnCl_2_, 500nM CuSO_4_, 1 μM ZnSO_4_, 200nM Na_2_MoO_4_, 10 μM NaCl, 10nM CoCl_2_, 50 μM FeSO_4_; pH adjusted to 5.7; supplemented with 1% (w/v) agar and 1% (w/v) sucrose; Haughn and Somerville, 1986] in a 16/8h light/dark regime at 21 °C.

*TS-GUS*, containing a transcriptionally silenced β-glucuronidase (*GUS*) transgene, has been described elsewhere (Morel *et al.*, 2000). All transgenic lines were generated in *A. thaliana* ecotype Col-0 using the floral dip method (Clough and Bent, 1998), unless indicated otherwise. The T-DNA insertion lines are displayed in Supplementary Fig. S1 at *JXB* online and were obtained from NASC (http://www.arabidopsis.info; McElver *et al.*, 2001; Alonso *et al.*, 2003). Primers used for genotyping mutant lines are summarized in Supplementary Table S1 at *JXB* online.

For generation of mutant combinations, single mutants were crossed, and confirmed F1 individuals were then used for further crossing into additional mutant lines. Segregation of the *TS-GUS* transgene was confirmed by GUS staining as described previously (Lang-Mladek *et al.*, 2010). The *idn2/fdm* hextuple and *idr* triple mutants were confirmed by genotyping in at least two independent lines each.

### Generation of constructs

For generation of *FDM1p::GUS*, we used primers 5′-GGGTCGA CAATGGGAGGACGGTGCTC-3′ and 5′- GGGTCGACCATCCT ACCTGCTTTACCTGG-3′ to amplify an *FDM1* promoter fragment (2001bp), which was subsequently cloned into pPZP-GUS (Diener *et al.*, 2000). A similar strategy was employed for *FDM2p::GUS* (primers 5′-GGGTCGACGCTACGCTCAAGCCTAATC-3′ and 5′-GGGTC GACCATCGTACCTGCTTTACCT-3′ to give a fragment of 2061bp) and for *FDM5p::GUS* (primers 5′- GGGTCGACGACTT CCCTTACGTCGCC-3′ and 5′-GGGTCGACCATCCTAGTAGGT GGCCTGT-3′ to give a fragment of 1670bp).

For the generation of *FDM* translational reporter constructs, we first amplified full-length cDNAs using primers 5′-GGCCCG GGGATGAGCATTTCTGATGAAGAG-3′ plus 5′-GGCCCGG GTCAGGTTCTTTTGCGTTTCAG-3′ for *FDM1*, and 5′-GGCC CGGGGATGGATAACAGTTCTGATG-3′ plus 5′-GGCCCGGG TTAGGCTCGTCTGCGTTTGAG-3′ for *FDM5*, and confirmed them by sequencing. Expression cassettes were cloned into a derivative of pPZP221 modified with the 35S terminator sequence (Hajdukiewicz *et al.*, 1994; Leitner *et al.*, 2012). For *FDM1–* and *FDM5*–VENUS reporters, a VENUS cassette was fused to the 3′ end of the respective coding regions of cDNAs, and the resulting constructs were either expressed under the control of the *RP40* promoter (Scheer *et al.*, 1997) or by endogenous *FDM* promoters (as was used for promoter–GUS constructs). For overexpression constructs, expression cassettes were cloned 3′ of an *RP40* promoter fragment, which was generated by PCR with primers 5′-GGGAATTCCTGGAGATATATCGGGTAAAGATGG-3′ and 5′-GGGAATTCTATCTCTCTTCTTCTTCTTCGCCGGGAA-3′.

### Expression analysis

For analysis of transcript levels, poly-A^+^-enriched RNA was isolated from different tissues as described previously (Sieberer *et al.*, 2003) and used for RT-PCR. GUS staining of *FDM* reporters and *TS-GUS* was performed as described previously (Lang-Mladek *et al.*, 2010). For determination of TS-GUS activity, we used established protocols (Morel *et al.*, 2000; Sieberer *et al.*, 2000). Briefly, soluble proteins from 12-d-old seedlings were extracted and incubated in 4-MUG assay buffer [50mM sodium phosphate, pH 7.0, 10mM β-mercaptoethanol, 10mM EDTA, 0.1 % (w/v) SDS,0.1% (v/v) Triton X-100, 1mM 4-methylumbelliferyl β-d-glucuronic acid (4-MUG)] at 37 °C. The reaction was stopped by adding 0.2M Na_2_CO_3_, and fluorescence was determined on a fluorescence spectrophotometer (Hitachi F-2000, excitation 365nm; emission 456nm). Emission values were normalized to the protein content of the samples.

For visualization of FDM–VENUS reporter lines, we used an SP2 Leica confocal laser-scanning microscope. Seedlings were either viewed alive after brief staining in propidium iodide (100 µg ml^–1^) to visualize cell boundaries or fixed in 3.7 % (v/v) formaldehyde in microtubule stabilization buffer (MTSB: 50mM PIPES, 5mM EGTA, 5mM MgSO_4_) for 15min. After three washes in MTSB, seedlings were mounted in MTSB containing 4,6-diamidine-2-phenylindole dihydrochloride (DAPI; 0.5ng ml^–1^) and viewed under the confocal laser-scanning microscope. For imaging, we used the following excitation conditions: 514nm (VENUS), 405nm (DAPI), and 561nm (propidium iodide).

### DNA extraction and methylation analysis

For comparative DNA methylation analysis, genomic DNA was extracted from 10-d-old seedlings (Dellaporta *et al.*, 1983). Digoxigenin-labelled probes were generated by PCR using primers 5′-GGATGCGATCATACCAG-3′ and 5′-GAGGGATGCAM CACSAG-3′ for the 5S rRNA gene or 5′-GTGGATATACCAAAAA CACAA-3′ and 5′-CTTAGCCTTCTTTTCAATCTCA-3′ for *AtMu1* (Zheng *et al.*, 2010). Oligonucleotides 5′-AAACCTTTCGTAAGCT ACAGCCACTTTGTT-3′ and 5′-TCGGATTGGTTCTTCCTACC TCTTTACCTT-3′ were used for the generation of a MEA-ISR probe (Cao and Jacobsen, 2002; Ausin *et al.*, 2009).

## Results

### Identification of XH/XS-domain protein loss-of-function alleles

For analysis of XH/XS-domain proteins, we first obtained potential loss-of-function mutants for all nine predicted members of the family, characterized by an XH and a closely related XS domain (Bateman, 2002). We adopted the nomenclature introduced recently (Xie *et al.*, 2012*a*; Zhang *et al.*, 2012), and characterized T-DNA insertion mutants in *FDM1*–*FDM5* and identified a potentially leaky T-DNA insertion line for *IDN2/RDM12* with an insertion in the 5′ untranslated region (Supplementary Fig. S1; Xie *et al.*, 2012*a*). Moreover, we obtained insertion mutants for all three additional loci predicted to encode proteins with an XH and an XS domain but to lack a zf-XS domain. Phylogenetic analyses performed with the entire predicted coding region of these loci highlighted an overall similarity to XH/XS-domain genes (Supplementary Fig. S1A). We therefore termed these loci *IDR1*–*IDR3* (for *IDN2 RELATED*; Supplementary Fig. S1). RT-PCR analysis of the insertion lines demonstrated defects in expression of full-length mRNAs for all lines except for *IDR2*, for which no transcript could be detected under our experimental conditions (Supplementary Fig. S1B). With respect to *idn2-3*, we found defects in expression of its 5′ untranslated region (Supplementary Fig. S1B). Nevertheless, earlier analysis of this *IDN2* allele suggested that it has retained some of its functionality (Xie *et al.*, 2012*a*)

Taken together, analysis of T-DNA insertion lines suggested identification of loss-of-function mutants for the XH/XS-domain loci, although we cannot rule out the possibility that the *idn2-3* and *idr2-1* alleles do not represent null alleles but still exhibit some remaining activity.

### Combinatorial loss of *IDN2*/*FDM*/*IDR* genes interferes with DNA methylation at selected RdDM target loci and antagonizes silencing of a *GUS* transgene

Analysis of *idn2/rdm12* mutant lines revealed defects in RdDM that appeared weaker than those of mutants deficient in additional, characterized components of the RdDM pathway (Ausin *et al.*, 2009; Zheng *et al.*, 2010). This led to suggestions of partially redundant activities among XH/XS-domain proteins, corroborated further by analysis of *fdm* mutant combinations (Xie *et al.*, 2012*a*). However, recent reports indicated only limited functional redundancy within the gene family, substantiated by the finding that IDN2 and at least two of its paralogues form complexes in which they could exhibit discernible functions in the control of RdDM (Ausin *et al.*, 2012; Xie *et al.*, 2012*b*; Zhang *et al.*, 2012).

Having identified potential loss-of-function lines for the *Arabidopsis* XH/XS-domain proteins, we determined their roles in DNA methylation at selected RdDM loci. Moreover, we obtained mutant combinations, which culminated in the generation of the *idn2-3 fdm1-1 idp2-1 fdm3-2 fdm4-2 fdm5-2* hextuple and the *idr1-1 idr2-1 idr3-1* triple mutant.

**Fig. 1.**
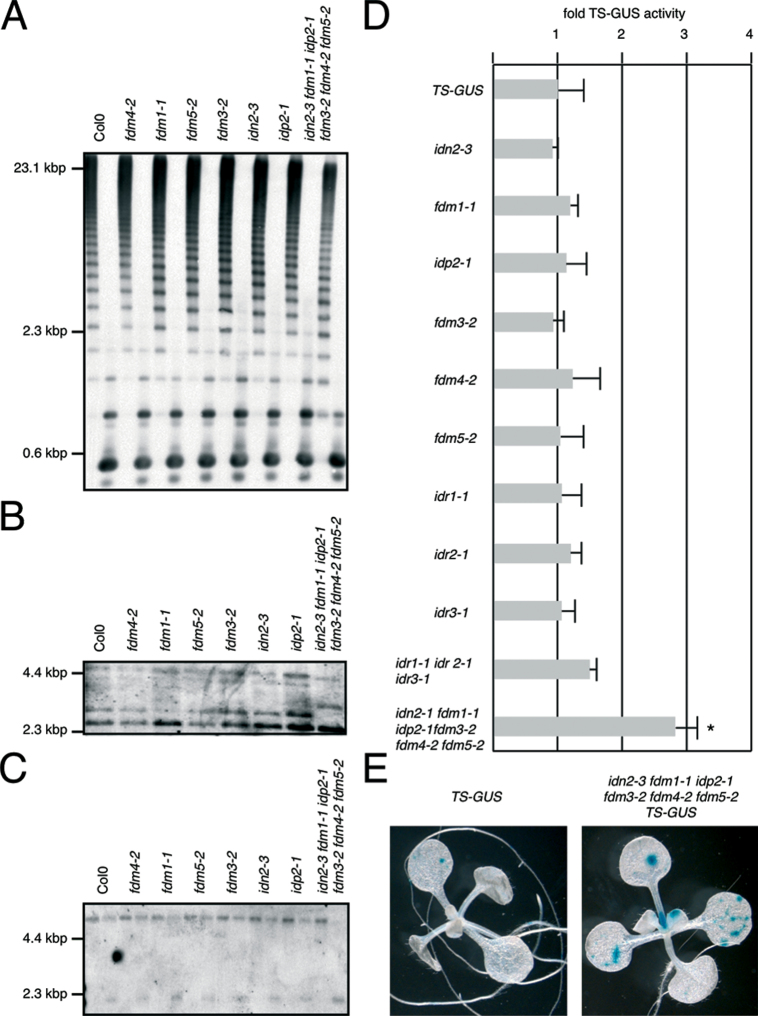
Analysis of DNA methylation and gene silencing in *idn2/fdm* mutants. (A) Southern blot performed with *Hpa*II-digested (left lanes) and *Hae*III-digested (right lanes) genomic DNA probed with the labelled 5S rRNA gene. Genotypes are indicated on top. (B) Southern blot performed with *Hae*III-digested genomic DNA probed with labelled *AtMU1* DNA. Genotypes are indicated on top. (C) Southern blot performed with *Hpa*II-cut (left lanes) and *Msp*I-cut (right lanes) genomic DNA that was probed for *MEA-ISR*. Genotypes are indicated on top. (D) *TS-GUS* activity in 14-d-old plantlets as determined by the 4-MUG assay. Activities are plotted as fold induction of activity found in the *TS-GUS* controls (given a value of 1). Standard deviations are indicated (*n*=4); the asterisk indicates a significant difference in reporter activity between *TS-GUS* and *idn2-3 fdm1-1 idp2-1 fdm3-2 fdm4-2 fdm5-2 TS-GUS* (*t*-test, *P*<0.05). (E) *TS-GUS* reporter activity in 14-d-old *TS-GUS* (left) and *idn2-3 fdm1-1 idp2-1 fdm3-2 fdm4-2 fdm5-2 TS-GUS* (right) plantlets. Blue staining indicates release of gene silencing.

Loss-of-function mutants affected in *IDN2* and *FDM* loci have been described to impact on DNA methylation of RdDM targets, and we therefore tested some of these loci (Zheng *et al.*, 2010). When probing methylation of the 5S rRNA gene by using restriction digests performed with *Hpa*II (for methylation analysis in the CG and CHG context) and with *Hae*III (for methylation analysis in the CHH context), a difference to that of the wild type was apparent in *idn2-3 fdm1-1 idp2-1 fdm3-2 fdm4-2 fdm5-2* DNA, indicative of weakly reduced CHG and CG methylation and a reduction in CHH methylation in the hextuple mutant (Fig. 1A). Single mutants as well as the *idr1-1 idr2-1 idr3-1* triple mutant combination exhibited no strong differences to wild-type DNA in comparison (Fig. 1A and Supplementary Fig. S2A). A similar observation was made when analysing the *AtMU1* locus in *Hae*III-cut DNA samples, with a reduction in DNA methylation in the CHH context apparent specifically for *idn2-3 fdm1-1 idp2-1 fdm3-2 fdm4-2 fdm5-2* DNA, whereas less pronounced or no differences were observed for the other mutant lines under our experimental conditions (Fig. 1B and Supplementary Fig. S2B). To further test the effects of XH/XS-domain proteins on methylation in the CG and CHG context, we analysed *Hpa*II- and *Msp*I-cut genomic DNA that was probed for MEA-ISR (Cao and Jacobsen, 2002). This suggested reduced symmetric DNA methylation most apparent in the *idn2-3 fdm1-1 idp2-1 fdm3-2 fdm4-2 fdm5-2* hextuple mutant (Fig. 1C). In contrast, we did not observe clear differences when analysing RdDM loci in *idr* single and *idr1-1 idr2-1 idr3-1* triple mutant lines, indicating that these loci are not essential for DNA methylation of analysed targets (Supplementary Fig. S2C).

Apart from well-established RdDM targets, we also analysed expression of the *TS-GUS* reporter line, containing a transcriptionally silenced *GUS* transgene that exhibits reactivation in a range of *Arabidopsis* gene-silencing mutants (Morel *et al.*, 2000; Elmayan *et al.*, 2005). Within this context, limited TS-GUS reactivation has been described for *Arabidopsis* mutants affected in loci functionally connected to RdDM (Elmayan *et al.*, 2005; Lang-Mladek *et al.*, 2010), and we asked whether or not XH/XS-domain proteins could be involved in the control of *TS-GUS* expression as well. *idn2-3 fdm1-1 idp2-1 fdm3-2 fdm4-2 fdm5-2 TS-GUS* exhibited a reproducible, statistically significant increase in TS-GUS activity when compared with *TS-GUS* controls (Fig. 1D, E). However, no prominent alterations in GUS activity were observed in any of the single mutants or in *idr1-1 idr2-1 idr3-1*. This indicated combinatorial activities of a subset of XH/XS-domain proteins in the regulation of *TS-GUS*.

Taken together, analysis of single mutant lines suggested only a limited impact on DNA methylation at endogenous RdDM target sites and activation of *TS-GUS*. In the case of *IDN2*, this might be a consequence of using a leaky allele, which would explain discrepancies between our results and findings made with potentially more severe *idn2* alleles (Ausin *et al.*, 2009; Finke *et al.*, 2012; Zhang *et al.*, 2012). Nevertheless, the more pronounced alterations in DNA methylation that were observed in the *idn2/fdm* hextuple mutant indicated that these loci exhibit overlapping, redundant activities in the control of DNA methylation. This corroborates findings by Xie *et al.* (2012*a*), who described additive effects on DNA methylation in *fdm* mutant combinations.

### *FDM* expression analysis

**Fig. 2.**
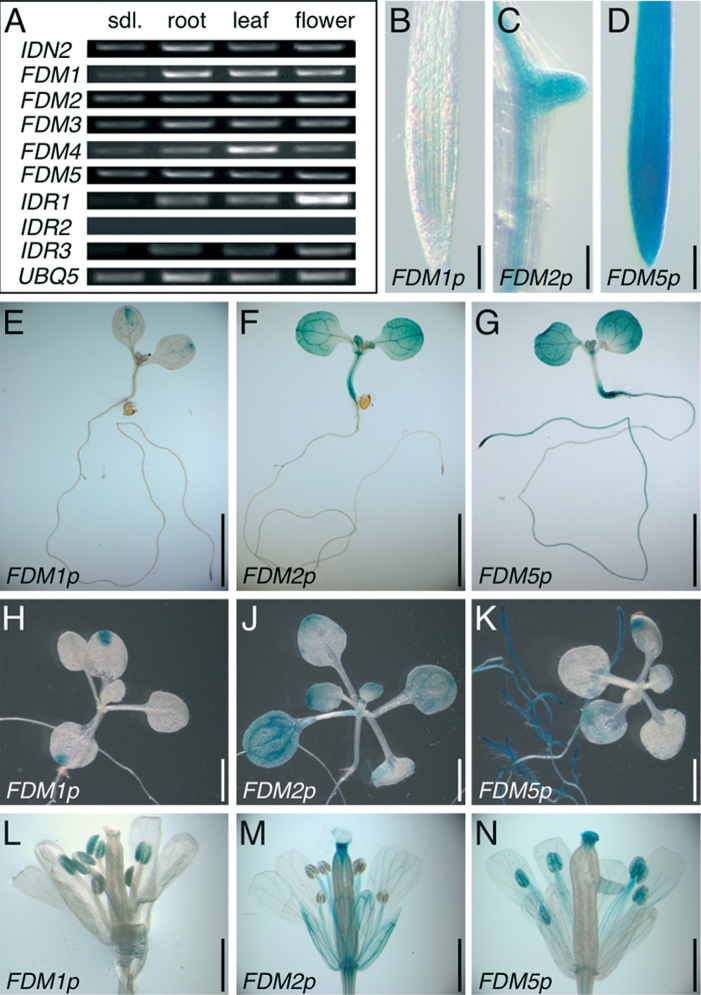
Expression analysis of XH/XS-domain genes in *Arabidopsis*. (A) RT-PCR performed with cDNA generated from seedling (sdl.), root, true leaf (leaf), and flower mRNA. *UQB5* transcript levels were used for standardization. (B–D) GUS staining of *FDM1p::GUS* (B), *FDM2p::GUS* (C), and *FDM5p::GUS* (D) in roots at 8 days after germination (DAG). *FDM2p::GUS* is predominantly active in the vasculature and emerging lateral roots (C). (E–G) GUS staining of *FDM1p::GUS* (B), *FDM2p::GUS* (C), and *FDM5p::GUS* (D) seedlings at 6 DAG. (H–K) GUS staining of *FDM1p::GUS* (H), *FDM2p::GUS* (J) and *FDM5p::GUS* (K) at 15 DAG. (L–N) GUS-stained flowers of *FDM1p::GUS* (L), *FDM2p::GUS* (M) and *FDM5p::GUS* (N) from 28-d-old plants. Bars, 75 μm (B, C); 5mm (E–G); 2.5mm (H–K); 1mm (L–N).

To determine expression of XH/XS-domain genes, we generated cDNAs from different plant tissues and analysed transcript levels by RT-PCR after normalization to *UBQ5* (Fig. 2A). These experiments revealed expression of all loci except for *IDR2*, transcripts of which were not detectable under our conditions. No prominent differences in tissue specificity in gene expression were observed when analysing *IDN2/RDM12* and *FDM* transcript levels in mRNA extracted from seedlings, roots, leaves, or flowers, whereas expression of *IDR1* and *IDR3* appeared most pronounced in floral organs (Fig. 2A).

**Fig. 3.**
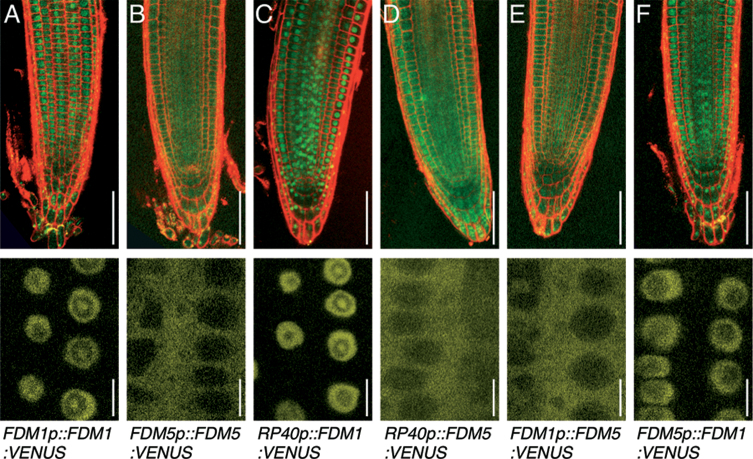
Subcellular localization of FDM–VENUS reporters (green, yellow) in live Col-0 root meristems (top panels) and in root meristem epidermis cells at higher magnification (bottom panels). (A) *FDM1p::FDM1:VENUS.* (B) *FDM5p::FDM5:VENUS.* (C) *FDM1p::FDM5:VENUS.* (D) *FDM5p::FDM1:VENUS.* (E) *RP40p::FDM1:VENUS.* (F) *RP40p::FDM5:VENUS.* Roots were counterstained with propidium iodide (100 µg ml^–1^, red) to visualize cell boundaries. Bars, 50 μm (top panels); 10 μm (bottom panels).

To address gene expression *in planta*, we generated promoter–*GUS* reporter constructs of selected *FDM* genes. Specifically, we analysed *FDM1*, *FDM2*, and *FDM5*, which represent a distinct subclade in the phylogenetic tree of the protein family (Supplementary Fig. S1A), in order to determine overlaps and differences in gene expression of these closely related loci. In seedlings, *FDM1p::GUS* staining was detectable in distal portions of cotyledons, and weaker expression was observed in root tips, a pattern that remained essentially unaltered throughout vegetative development (Fig. 2B, E, H). Additional *FDM1p::GUS* activity was observed in flowers, where it appeared to be restricted to the male gametophyte (Fig. 2L). Only weak *FDM1p::GUS* activity was observed in root meristems, which differed from RT-PCR analyses and root expression of a *VENUS*-tagged *FDM1* translational reporter line (Figs 2A, B, and 3), suggesting that *cis*-acting elements required for *FDM1* expression in root tissue are not fully active in *FDM1p::GUS*. *FDM2p::GUS* and *FDM5p::GUS* expression was stronger in comparison, exhibiting signals in roots, hypocotyls, and cotyledons of seedlings (Fig. 2C, D, F, G), whereas true leaves showed strong expression only of *FDM2p::GUS* (Fig. 2J). In flowers, *FDM2p::GUS* staining produced signals in the vasculature of sepals and petals as well as in anther filaments and parts of the style (Fig. 2M), whereas *FDM5p::GUS* staining was visible in the anthers, including pollen as well as in the stigma (Fig. 2N). In addition, *FDM5p::GUS* showed activity in the entire root, both at the seedling stage and in older plants (Fig. 2D, G), whereas FDM2::GUS staining was essentially restricted to the root vasculature and emerging lateral roots (Fig. 2C).

Taken together, the results obtained from gene expression and reporter analysis indicated distinct gene expression patterns, reflecting tissue specificity in activity of these closely related *Arabidopsis* XH/XS-domain genes.

To determine the subcellular localization of FDM proteins, we generated *FDM1:VENUS* and *FDM5:VENUS* expression cassettes, in which the entire FDM-coding regions were fused to the VENUS fluorescent protein (Nagai *et al.*, 2002), and expressed under control of their endogenous promoters. Confocal laser-scanning microscopy analysis revealed that both *FDM1p::FDM1:VENUS* and *FDM5p::FDM5:VENUS* expression could be observed in root meristems, but the signals differed in their subcellular localization. When expressed in Col-0, *FMD1p::FDM1:VENUS* lines accumulated signals in the nucleus, whereas *FDM5p::FDM5:VENUS* exhibited signals predominantly in the cytoplasm (Fig. 3A, B).

We then asked whether or not differences in FDM reporter localization could result from differences in expression levels, and fused both FDM–VENUS expression cassettes to the strong ribosomal protein *RP40* promoter (Scheer *et al.*, 1997) and analysed expression of the resultant reporter constructs in wild-type Col-0. Ectopic overexpression of both transgenes confirmed the results obtained with endogenous reporters, with *RP40p::FDM1:VENUS* root meristems exhibiting signals in nuclei, whereas *RP40p::FDM5:VENUS* signals were most pronounced in the cytoplasm (Fig. 3C, D). In additional experiments, we performed promoter swaps and expressed *FDM1:VENUS* under the control of the *FDM5* promoter and vice versa, expressing *FDM5:VENUS* by the *FDM1* promoter. These experiments demonstrated nuclear signals in *FDM5p::FDM1:VENUS* lines and cytoplasmic signals in *FDM1p::FDM5:VENUS* lines (Fig. 3E, F). Collectively, our analyses indicated that neither variations in expression domains (promoter swaps) nor expression levels (overexpression) significantly affected the distinct subcellular distribution of the FDM1 and FDM5 reporter proteins. This is in agreement with roles for FDM proteins in both the nucleus and the cytoplasm.

### Relocation of FDM1–VENUS coincides with defects in XH/XS-domain proteins

**Fig. 4.**
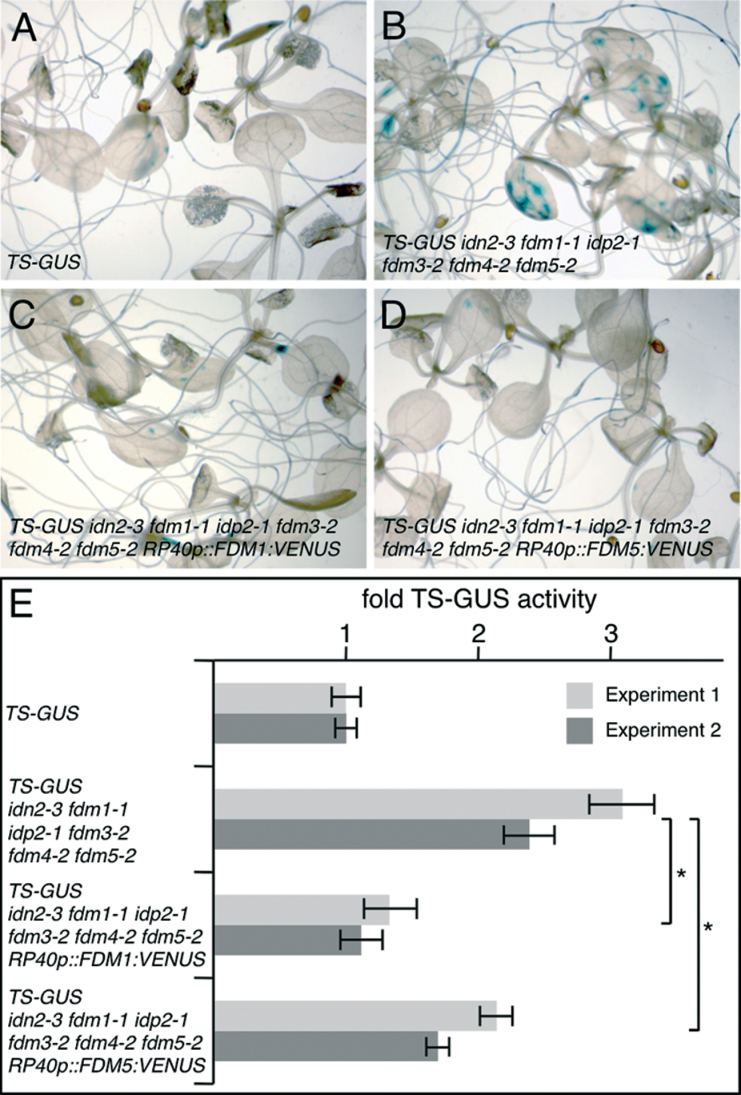
Functional analysis of FDM–VENUS reporter proteins. (A–D) TS-GUS staining in 10-d-old TS-GUS (A), *idn2-3 fdm1-1 idp2-1 fdm3-2 fdm4-2 fdm5-2 TS-GUS* (B), *idn2-3 fdm1-1 idp2-1 fdm3-2 fdm4-2 fdm5-2 TS-GUS RP40p::FDM1:VENUS* (C), and *idn2-3 fdm1-1 idp2-1 fdm3-2 fdm4-2 fdm5-2 TS-GUS RP40p::FDM5:VENUS* (D). (E) TS-GUS activity in 14-d-old plantlets as determined by a 4-MUG assay. Activities are plotted as fold induction of activity found in the *TS-GUS* controls (given a value of 1). Two independent experiments are shown and standard deviation is indicated. Asterisks and brackets highlight significant differences in *TS-GUS* activity between samples (*t*-test; *P*<0.05).

Differences in the subcellular distribution of closely related FDM proteins are suggestive of differing functions. We further addressed this issue, and first determined FDM reporter protein functionality in the *idn2/fdm* mutant background. To this end, we introduced *RP40p::FDM1:VENUS* and *RP40p::FDM5:VENUS* into *idn2-3 fdm1-1 idp2-1 fdm3-2 fdm4-2 fdm5-2* expressing the *TS-GUS* transgene. TS-GUS activity was increased in *idn2-3 fdm1-1 idp2-1 fdm3-2 fdm4-2 fdm5-2*, reflecting defects in controlling the silenced state of the transgene (Figs 1D, E, and 4A, B, E). This was different in *idn2-3 fdm1-1 idp2-1 fdm3-2 fdm4-2 fdm5-2 TS-GUS RP40p::FDM1:VENUS* and *idn2-3 fdm1-1 idp2-1 fdm3-2 fdm4-2 fdm5-2 TS-GUS RP40p::FDM5:VENUS*, which both exhibited reduced TS-GUS activity, a response that appeared more pronounced upon expression of the *RP40p::FDM1:VENUS* reporter construct (Fig. 4C, D, E). These findings suggested that both FDM–VENUS reporter proteins are capable of rescuing defects in *TS-GUS* silencing (Fig. 4C, D).

**Fig. 5.**
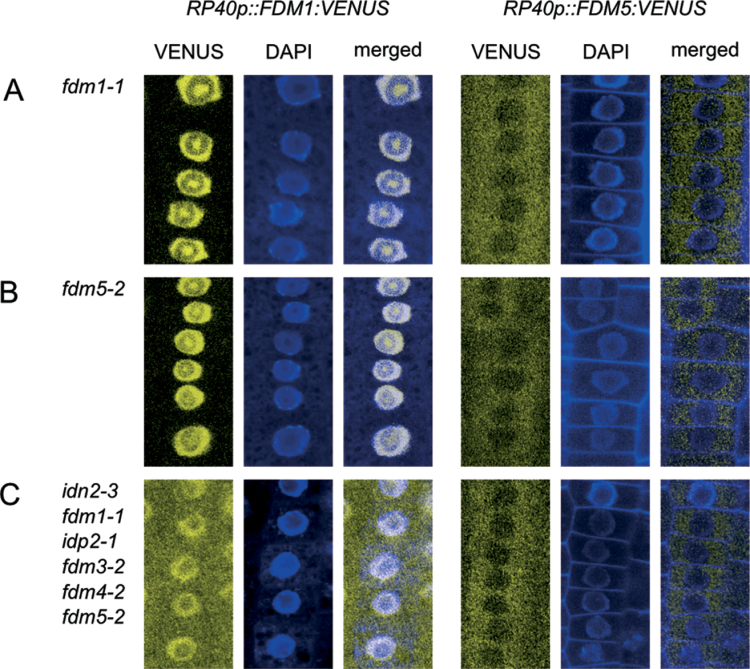
Subcellular localization of FDM–VENUS reporter proteins (yellow) in fixed root meristem cells of *idn2/fdm* mutants. (A) *RP40p::FDM1:VENUS* (left panels) and *RP40p::FDM5:VENUS* (right panels) in *fdm1-1*. Note the accumulation of FDM1–VENUS reporter signals in the nucleolar cavity, whilst weak signals only are found in the surrounding dense fibrillar zone. (B) *RP40p::FDM1:VENUS* (left panels) and *RP40p::FDM5:VENUS* (right panels) in *fdm5-2*. (C) *RP40p::FDM1:VENUS* (left panels) and *RP40p::FDM5:VENUS* (right panels) in *idn2-3 fdm1-1 idp2-1 fdm3-2 fdm4-2 fdm5-2*. Seedlings were fixed in formaldehyde (3.7%, v/v) and counterstained with DAPI (0.5ng ml^–1^, blue) to visualize nuclei. Bars,10 μm.

Analysis of FDM–VENUS reporters in Col-0 revealed distinct FDM1–VENUS and FDM5–VENUS signals in root meristem cells (Fig. 3). On the other hand, both reporters rescued defects in *TS-GUS* expression control in *idn2-3 fdm1-1 idp2-1 fdm3-2 fdm4-2 fdm5-2* at least to some extent (Fig. 4), raising questions about the mechanisms involved. To address this issue, we assayed the FDM–VENUS reporter localization in the *idn2/fdm* mutant background. We first analysed *RP40p::FDM1:VENUS* and *RP40p::FDM5:VENUS* expression in the corresponding *fdm1-1* and *fdm5-2* single mutants. Similar to Col-0, we found FDM1:VENUS signals in the nucleus of *fdm1-1* root meristem cells, whereas FDM5:VENUS was visible in the cytoplasm of *fdm5-2* root meristem cells (Fig. 5A). Identical results were obtained when analysing FDM1:VENUS distribution in *fdm5-2* and FDM5:VENUS distribution in *fdm1-1* (Fig. 5B), suggesting that defects in endogenous *FDM1* and *FDM5* do not influence FDM–VENUS reporter distribution.

We then expressed *RP40p::FDM1:VENUS* and *RP40p::FDM5:VENUS* in the *idn2-3 fdm1-1 idp2-1 fdm3-2 fdm4-2 fdm5-2* hextuple mutant and determined reporter localization in root meristem cells. Subcellular distribution of FDM5:VENUS remained detectable predominantly in the cytoplasm (Fig. 5C), but alterations were observed for *RP40p::FDM1:VENUS*. This reporter line exhibited signals in the nuclei and cytoplasm when expressed in *idn2-3 fdm1-1 idp2-1 fdm3-2 fdm4-2 fdm5-2* (Fig. 5C). Thus, unlike in Col-0, *fdm1-1*, or *fdm5-2*, in which FDM1:VENUS signals are restricted to the nucleus, a combinatorial loss of XH/XS-domain proteins coincided with a visibly altered subcellular localization of FDM1, no longer confined to the nuclear compartment.

When taken together, analysis of *FDM* reporter lines indicated localization in the nucleus and cytoplasm and highlighted flexible subcellular FDM protein distribution under conditions when cytoplasmic FDM protein abundance appeared restricted.

## Discussion

Gene amplification by duplication is a common phenomenon observed in virtually all classes of organisms. Plants, in particular, have evolved numerous gene families of variable sizes, which allows evolution of modified gene functions but also represents a resource for redundant gene activities, resulting in an increased genetic robustness. In the case of *Arabidopsis IDN2/RDM12* and its closely related paralogues, both mechanisms seem to be in effect, supported by experimental evidence for neofunctionalization but also for redundant activities within this gene family (Ausin *et al.*, 2009; Zheng *et al.*, 2010; Ausin *et al.*, 2012; Xie *et al.*, 2012*a*, *b*; Zhang *et al.*, 2012).

Characterization of *IDN2/RDM12* demonstrated rather mild defects in siRNA biogenesis and DNA methylation control, which led to the suggestion of redundant gene function (Ausin *et al.*, 2009; Zheng *et al.*, 2010). This is supported by the work by Xie *et al.* (2012*a*), demonstrating synergistic effects in some *idn2/fdm* and *fdm* double and triple mutant combinations. Currently, it cannot be excluded that some of the *idn2/fdm* alleles in use have retained some of their functionality. For example, *idn2-3* has been described to represent a leaky allele (Xie *et al.*, 2012*a*), which makes it difficult to determine the contribution of individual loci in mutant combinations. Nevertheless, the phenotype of the *idn2-3 fdm1-1 idp2-1 fdm3-2 fdm4-2 fdm5-2* hextuple mutant is clearly consistent with a model suggesting overlapping functions for at least some of the XH/XS-domain genes, as it exhibited defects in DNA methylation and transgene silencing that were more pronounced than in any of the corresponding single mutants.

Analysis of the *idr* mutants did not reveal alterations in DNA methylation at selected RdDM target sites. This suggests roles for *IDR* genes, either different from, or only partially overlapping with, the activities of the *IDN2* and *FDM* loci. Homology-based searches revealed that IDN2/FDM and IDR proteins differ in their N-terminal portion, as there is no zf-XS domain predicted for IDR proteins. Differing DNA methylation phenotypes observed in *idn2/fdm* and *idr* mutant combinations thus could indicate a prominent function for the zf-XS domain in the regulation of RdDM, but further experiments are required to characterize this domain.

Recent reports provided evidence for divergent functions of *IDN2* and some of its paralogues (Ausin *et al.*, 2012; Xie *et al.*, 2012*b*; Zhang *et al.*, 2012). Specifically, analysis of *idn2 fdm1 fdm2* mutant combinations have suggested non-redundant roles for *IDN2* and *FDM1/FDM2* (Ausin *et al.*, 2012). Strong experimental support for a functional diversification of XH/XS-domain proteins comes from observations demonstrating that IDN2 as well as FDM1 have the ability to form homo- and heteromeric protein complexes, in which monomers might carry out different functions (Ausin *et al.*, 2012; Xie *et al.*, 2012*b*; Zhang *et al.*, 2012). Another report demonstrated a physical interaction between IDN2/RDM12 and a subunit of a SWI/SNF ATP-dependent nucleosome-remodelling complex, possibly involved in mediating the effects of long non-coding RNAs on the epigenetic status of chromatin (Zhu *et al.*, 2013). It thus appears that variations in configuration and composition of XH/XS-domain protein complexes might reflect distinct roles for these proteins, depending on the type of complex being formed. Our analysis of FDM reporter proteins provides additional evidence for distinct roles of FDM proteins, indicated by the prominent differences in their subcellular localization.

XH/XS-domain family genes have been implicated to act in a late step of the RdDM pathway, with XH/XS-domain protein complexes potentially required for stabilization of AGO4-delivered siRNAs when paired to PollV transcripts at chromatin sites (Ausin *et al.*, 2012; Finke *et al.*, 2012). Furthermore, IDN2 has been suggested to guide an *Arabidopsis* SWI/SNF remodelling complex to genomic loci in the process of gene silencing (Zhu *et al.*, 2013). All these functions would be in agreement with a nuclear localization of XH/XS-domain proteins as we observed for FDM1–VENUS reporters. The cytoplasmic localization of FDM5–VENUS reporters highlights additional functions for XH/XS-domain proteins apparently detached from their activities at chromatin. Apart from that, FDM1, which otherwise exhibits strictly nuclear localization, appears to relocalize to the cytoplasm under conditions when availability of further FDMs is limited, further suggestive of an essential role for cytoplasmic FDM localization.

The *Arabidopsis* XS-domain protein SGS3 appears to function in the cytoplasm, and is suggested to be involved in the generation or maturation of viral siRNAs (Mourrain *et al.*, 2000; Peragine *et al.*, 2004; Borsani *et al.*, 2005; Hoffer *et al.*, 2011). In addition, Ye *et al.* (2012) demonstrated that loading of siRNA onto AGO4 occurred in the cytoplasm, and provided evidence suggesting that such complex assembly triggers its subsequent relocation to the nucleus. This finding indicated that mechanisms controlling RdDM are not entirely restricted to the nucleus. A similar scenario could be envisioned for XH/XS-domain proteins, in which cytoplasmic localization of FDM5 and possibly further members of the protein family might reflect involvement in early steps of the RdDM pathway. This could, for example, concern stabilization of AGO/siRNA complex formation in the cytoplasm, as well as potential roles in guidance of such complexes to destination sites in the nucleus. Clearly, further experiments are required to characterize these elusive activities.

Our experiments demonstrated that FDM1-reporter relocation coincided with defects in additional members of the gene family. This observation suggests that FDM1, whilst otherwise exhibiting nuclear localization, could adopt activities in the cytoplasm. XH/XS-domain proteins have been demonstrated to form multimeric complexes (Ausin *et al.*, 2012; Xie *et al.*, 2012*b*; Zhang *et al.*, 2012), and there is a possibility that some of these complexes might function in the cytoplasm. It is possible that increased cytoplasmic accumulation of FDM1 results from its relocation as part of a protein complex, in which it would take over the function of cytoplasmic FDM proteins like FDM5. This might explain FDM1–VENUS relocation occurring only in the severe *idn2-3 fdm1-1 idp2-1 fdm3-2 fdm4-2 fdm5-2* hextuple mutant. In an alternate scenario, loss of additional XH/XS-domain proteins in the *idn2-3 fdm1-1 idp2-1 fdm3-2 fdm4-2 fdm5-2* mutant might interfere with correct nuclear relocation of otherwise cytoplasmic FDM1. A role for XH/XS proteins in the cytoplasm is supported by the distribution of functional FDM5–VENUS, which accumulates predominantly outside the nucleus, underlining a scenario in which, apart from their activity in association with chromatin domains, cytoplasmic localization of XH/XS-domain proteins contributes to their function in the control of DNA methylation.

Overall, our findings suggest activities of XH/XS proteins in both the nucleus and cytoplasm, and it seems possible that relocation of FDM proteins between these compartments could account for partially redundant activities within this gene family. It will be interesting to study the principles and mechanisms of this potentially highly versatile strategy to compensate for activities within functionally diverging gene families.

## Supplementary Material

Supplementary Data

## Abbreviations:

4-MUG: 4-methylumbelliferyl β-d-glucuronic acid
RdDM: RNA-directed DNA methylation
siRNA: small interfering RNA
zf: zinc finger.

## References

[CIT0001] Alonso JM Stepanova AN Leisse TJ 2003 Genome-wide insertional mutagenesis of *Arabidopsis thaliana* . Science 301, 653–657 12893945 10.1126/science.1086391

[CIT0002] Ausin I Greenberg MV Simanshu DK 2012 INVOLVED IN DE NOVO 2-containing complex involved in RNA-directed DNA methylation in *Arabidopsis* . Proceedings of the National Academy of Sciences, USA 109, 8374–8381 10.1073/pnas.1206638109 PMC3365198 22592791

[CIT0003] Ausin I Mockler TC Chory J Jacobsen SE 2009 IDN1 and IDN2 are required for *de novo* DNA methylation in *Arabidopsis thaliana* . Nature Structural and Molecular Biology 16, 1325–1327 10.1038/nsmb.1690 PMC2842998 19915591

[CIT0004] Bateman A 2002 The SGS3 protein involved in PTGS finds a family. BMC Bioinformatics 3, 21 12162795 10.1186/1471-2105-3-21 PMC119857

[CIT0005] Borsani O Zhu J Verslues PE Sunkar R Zhu JK 2005 Endogenous siRNAs derived from a pair of natural *cis*-antisense transcripts regulate salt tolerance in *Arabidopsis* . Cell 123, 1279–1291 16377568 10.1016/j.cell.2005.11.035 PMC3137516

[CIT0006] Cannon SB Mitra A Baumgarten A Young ND May G 2004 The roles of segmental and tandem gene duplication in the evolution of large gene families in *Arabidopsis thaliana* . BMC Plant Biology 4, 10 15171794 10.1186/1471-2229-4-10 PMC446195

[CIT0007] Cao X Jacobsen SE 2002 Locus-specific control of asymmetric and CpNpG methylation by the DRM and CMT3 methyltransferase genes. Proceedings of the National Academy of Sciences, USA 99 (Suppl. 4), 16491–16498 10.1073/pnas.162371599 PMC139913 12151602

[CIT0008] Clough SJ Bent AF 1998 Floral dip: a simplified method for *Agrobacterium*-mediated transformation of *Arabidopsis thaliana* . The Plant Journal 16, 735–743 10069079 10.1046/j.1365-313x.1998.00343.x

[CIT0009] Dellaporta SL Wood J Hicks JB 1983 A plant DNA miniprepration: version II. Plant Molecular Biology Reporter 1, 19–21

[CIT0010] Diener AC Li H Zhou W Whoriskey WJ Nes WD Fink GR 2000 Sterol methyltransferase 1 controls the level of cholesterol in plants. Plant Cell 12, 853–870 10852933 10.1105/tpc.12.6.853 PMC149089

[CIT0011] Elmayan T Adenot X Gissot L Lauressergues D Gy I Vaucheret H 2009 A neomorphic *sgs3* allele stabilizing miRNA cleavage products reveals that SGS3 acts as a homodimer. FEBS Journal 276, 835–844 19143842 10.1111/j.1742-4658.2008.06828.x

[CIT0012] Elmayan T Proux F Vaucheret H 2005 Arabidopsis RPA2: a genetic link among transcriptional gene silencing, DNA repair, and DNA replication. Current Biology 15, 1919–1925 16271868 10.1016/j.cub.2005.09.044

[CIT0013] Finke A Kuhlmann M Mette MF 2012 IDN2 has a role downstream of siRNA formation in RNA-directed DNA methylation. Epigenetics 7, 950–960 22810086 10.4161/epi.21237 PMC3427290

[CIT0014] Gasciolli V Mallory AC Bartel DP Vaucheret H 2005 Partially redundant functions of *Arabidopsis* DICER-like enzymes and a role for DCL4 in producing *trans*-acting siRNAs. Current Biology 15, 1494–1500 16040244 10.1016/j.cub.2005.07.024

[CIT0015] Gottlieb LD 2003 Plant polyploidy: gene expression and genetic redundancy. Heredity 91, 91–92 12886270 10.1038/sj.hdy.6800317

[CIT0016] Hajdukiewicz P Svab Z Maliga P 1994 The small, versatile pPZP family of Agrobacterium binary vectors for plant transformation. Plant Molecular Biology 25, 989–994 7919218 10.1007/BF00014672

[CIT0017] Haughn GW Somerville C 1986 Sulfonylurea-resistant mutants of *Arabidopsis thaliana* . Molecular and General Genetics 204, 430–434

[CIT0018] Hoffer P Ivashuta S Pontes O Vitins A Pikaard C Mroczka A Wagner N Voelker T 2011 Posttranscriptional gene silencing in nuclei. Proceedings of the National Academy of Sciences, USA 108, 409–414 10.1073/pnas.1009805108 PMC3017132 21173264

[CIT0019] Katiyar-Agarwal S Morgan R Dahlbeck D Borsani O Villegas A Jr. Zhu JK Staskawicz BJ Jin H 2006 A pathogen-inducible endogenous siRNA in plant immunity. Proceedings of the National Academy of Sciences, USA 103, 18002–18007 10.1073/pnas.0608258103 PMC1693862 17071740

[CIT0020] Lang-Mladek C Popova O Kiok K Berlinger M Rakic B Aufsatz W Jonak C Hauser MT Luschnig C 2010 Transgenerational inheritance and resetting of stress-induced loss of epigenetic gene silencing in *Arabidopsis* . Molecular Plant 3, 594–602 20410255 10.1093/mp/ssq014 PMC2877484

[CIT0021] Leitner J Petrasek J Tomanov K Retzer K Parezova M Korbei B Bachmair A Zazimalova E Luschnig C 2012 Lysine63-linked ubiquitylation of PIN2 auxin carrier protein governs hormonally controlled adaptation of Arabidopsis root growth. Proceedings of the National Academy of Sciences, USA 109, 8322–8327 10.1073/pnas.1200824109 PMC3361439 22556266

[CIT0022] Lynch M Force A 2000 The probability of duplicate gene preservation by subfunctionalization. Genetics 154, 459–473 10629003 10.1093/genetics/154.1.459 PMC1460895

[CIT0023] McElver J Tzafrir I Aux G 2001 Insertional mutagenesis of genes required for seed development in *Arabidopsis thaliana* . Genetics 159, 1751–1763 11779812 10.1093/genetics/159.4.1751 PMC1461914

[CIT0024] Morel JB Mourrain P Beclin C Vaucheret H 2000 DNA methylation and chromatin structure affect transcriptional and post-transcriptional transgene silencing in *Arabidopsis* . Current Biology 10, 1591–1594 11137011 10.1016/s0960-9822(00)00862-9

[CIT0025] Mourrain P Beclin C Elmayan T 2000 *Arabidopsis SGS2* and *SGS3* genes are required for posttranscriptional gene silencing and natural virus resistance. Cell 101, 533–542 10850495 10.1016/s0092-8674(00)80863-6

[CIT0026] Nagai T Ibata K Park ES Kubota M Mikoshiba K Miyawaki A 2002 A variant of yellow fluorescent protein with fast and efficient maturation for cell-biological applications. Nature Biotechnology 20, 87–90 10.1038/nbt0102-87 11753368

[CIT0027] Nowak MA Boerlijst MC Cooke J Smith JM 1997 Evolution of genetic redundancy. Nature 388, 167–171 9217155 10.1038/40618

[CIT0028] Ohno S 1970 Evolution by gene duplication. New York: Springer Verlag

[CIT0029] Peragine A Yoshikawa M Wu G Albrecht HL Poethig RS 2004 *SGS3* and *SGS2*/*SDE1*/*RDR6* are required for juvenile development and the production of *trans*-acting siRNAs in *Arabidopsis* . Genes and Development 18, 2368–2379 15466488 10.1101/gad.1231804 PMC522987

[CIT0030] Qian W Liao BY Chang AY Zhang J 2010 Maintenance of duplicate genes and their functional redundancy by reduced expression. Trends in Genetics 26, 425–430 20708291 10.1016/j.tig.2010.07.002 PMC2942974

[CIT0031] Qin Y Ye H Tang N Xiong L 2009 Systematic identification of X1-homologous genes reveals a family involved in stress responses in rice. Plant Molecular Biology 71, 483–496 19701685 10.1007/s11103-009-9535-5

[CIT0032] Scheer I Ludevid MD Regad F Lescure B Pont-Lezica RF 1997 Expression of a gene encoding a ribosomal p40 protein and identification of an active promoter site. Plant Molecular Biology 35, 905–913 9426609 10.1023/a:1005956601270

[CIT0033] Sieberer T Hauser MT Seifert GJ Luschnig C 2003 *PROPORZ1*, a putative *Arabidopsis* transcriptional adaptor protein, mediates auxin and cytokinin signals in the control of cell proliferation. Current Biology 13, 837–842 12747832 10.1016/s0960-9822(03)00327-0

[CIT0034] Sieberer T Seifert GJ Hauser MT Grisafi P Fink GR Luschnig C 2000 Post-transcriptional control of the *Arabidopsis* auxin efflux carrier EIR1 requires AXR1. Current Biology 10, 1595–1598 11137012 10.1016/s0960-9822(00)00861-7

[CIT0035] Vaucheret H 2008 Plant ARGONAUTES. Trends in Plant Science 13, 350–358 18508405 10.1016/j.tplants.2008.04.007

[CIT0036] Xie M Ren G Costa-Nunes P Pontes O Yu B 2012a A subgroup of SGS3-like proteins act redundantly in RNA-directed DNA methylation. Nucleic Acids Research 40, 4422–4431 22302148 10.1093/nar/gks034 PMC3378875

[CIT0037] Xie M Ren G Zhang C Yu B 2012b The DNA- and RNA-binding protein FACTOR of DNA METHYLATION 1 requires XH domain-mediated complex formation for its function in RNA-directed DNA methylation. The Plant Journal 72, 491–500 22757778 10.1111/j.1365-313X.2012.05092.x

[CIT0038] Ye R Wang W Iki T Liu C Wu Y Ishikawa M Zhou X Qi Y 2012 Cytoplasmic assembly and selective nuclear import of *Arabidopsis* ARGONAUTE4/siRNA complexes. Molecular Cell 46, 859–870 22608924 10.1016/j.molcel.2012.04.013

[CIT0039] Zhang CJ Ning YQ Zhang SW Chen Q Shao CR Guo YW Zhou JX Li L Chen S He XJ 2012 IDN2 and its paralogs form a complex required for RNA-directed DNA methylation. PLoS Genetics 8, e1002693 22570638 10.1371/journal.pgen.1002693 PMC3342958

[CIT0040] Zheng Z Xing Y He XJ Li W Hu Y Yadav SK Oh J Zhu JK 2010 An SGS3-like protein functions in RNA-directed DNA methylation and transcriptional gene silencing in Arabidopsis. The Plant Journal 62, 92–99 20059743 10.1111/j.1365-313X.2010.04130.x PMC2858770

[CIT0041] Zhu Y Rowley MJ Bohmdorfer G Wierzbicki AT 2013 A SWI/SNF chromatin-remodeling complex acts in noncoding RNA-mediated transcriptional silencing. Molecular cell 49, 298–309 23246435 10.1016/j.molcel.2012.11.011 PMC3560041

