# Supplementary material for: Expression analysis of Arabidopsis XH/XS-domain proteins indicates overlapping and distinct functions for members of this gene family
Source: J Exp Bot. 2014 Feb 18;65(4):1217–27. doi: 10.1093/jxb/ert480 (PMC3935573; doi:10.1093/jxb/ert480)
Supplement: Supplementary Data [file supp_65_4_1217__index.html]

Expression analysis of Arabidopsis XH/XS-domain proteins indicates overlapping and distinct functions for members of this gene family — Supplementary Data 

# Expression analysis of *Arabidopsis* XH/XS-domain proteins indicates overlapping and distinct functions for members of this gene family

## Supplementary Data

Data files

**Files in this Data Supplement:**

- Supplementary Data - Supplementary Data
