# Supplementary material for: Expression analysis of Arabidopsis XH/XS-domain proteins indicates overlapping and distinct functions for members of this gene family
Source: J Exp Bot. 2014 Feb 18;65(4):1217–27. doi: 10.1093/jxb/ert480 (PMC3935573; doi:10.1093/jxb/ert480)
Supplement: Supplementary Data [file supp_ert480_jexbot116012_file001.pdf]

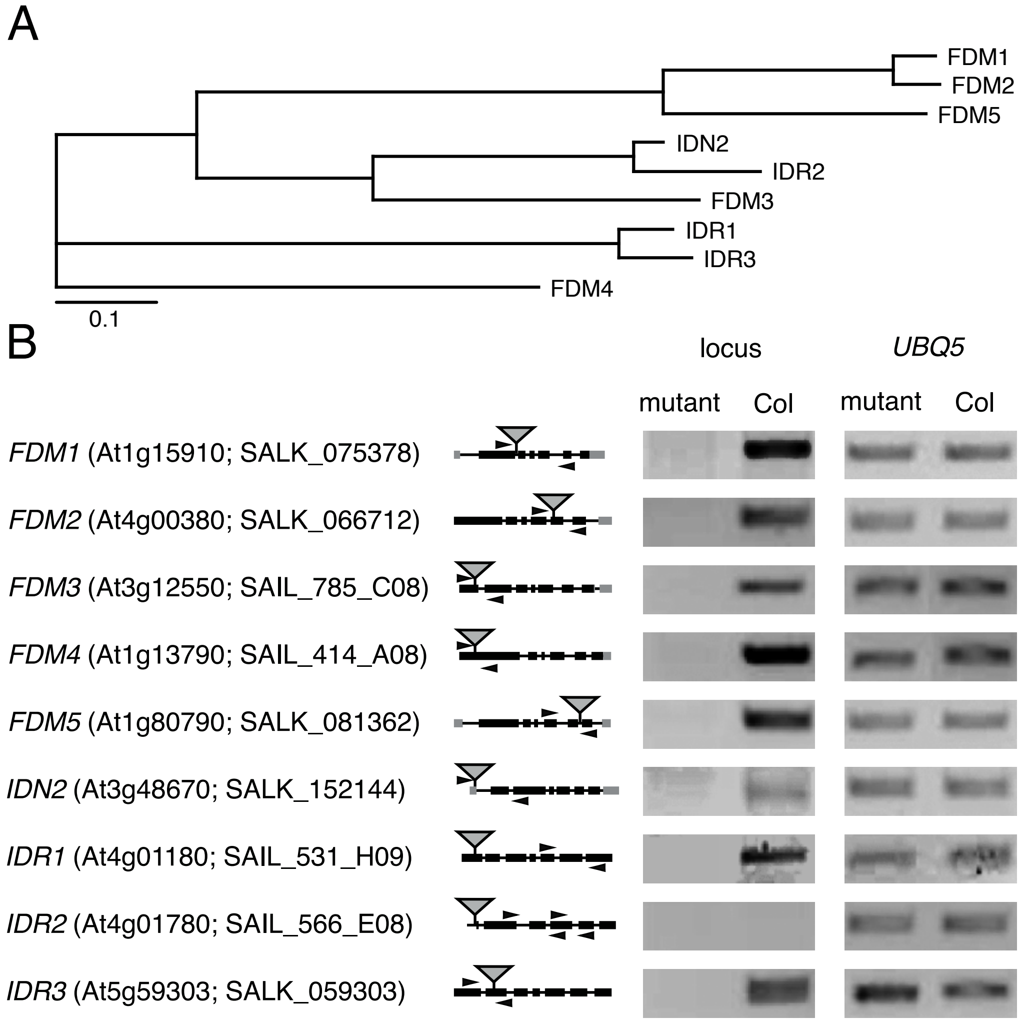

Butt et al., Fig. S1

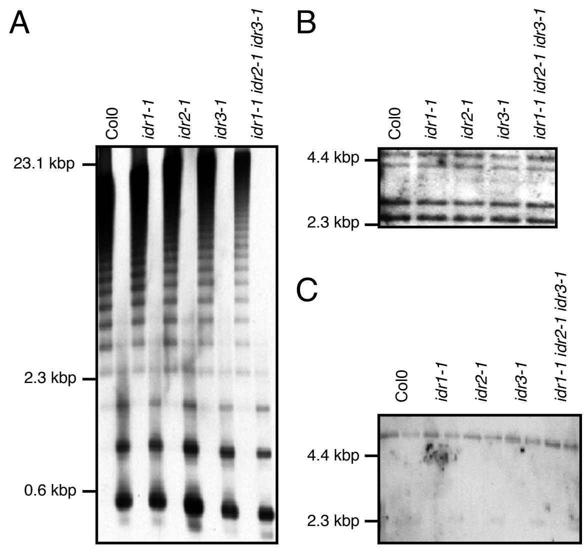

Butt et al., Fig. S2

Butt et al.; Supplemental Information

Fig. S1: Phylogeny of Arabidopsis XH/XS-domain proteins, and molecular characterization of *idn2/fdm* and *idr* loss-of-function alleles. **A)** Phylogenetic tree of *Arabidopsis* IDN2/FDM and IDR predicted proteins. Multiple sequence alignments were performed with ClustalX (Larkin *et al.*, 2007) by using default settings. The resulting alignment was used for generation of a neighborhood-joining tree, corrected for multiple substitutions in the alignment and an exclusion of gapped positions. Branch lengths are proportional to the number of substitutions per site (see scale bar). **B)** PCR-characterization of *idn2/fdm* and *idr* loss-of-function alleles. Left column: Loci together with gene identifiers and T-DNA insertion lines used in this study (in brackets). Right column: RT-PCR with locus-specific primers (locus) flanking respective T-DNA insertion sites performed with mutant and Col-0 cDNAs. As a control, *UBQ5* was amplified from all cDNA samples used. Small arrowheads indicate the position of primers used for expression analysis.

Fig. S2: Analysis of DNA methylation and gene silencing in *idr* mutants. **A)** Southern blot performed with *Hpa*II- (left lanes) and *Hae*III-(right lanes)-digested genomic DNA probed with labeled 5S-rDNA. Genotypes are indicated on top. **B)** Southern blot performed with *Hae*III-digested genomic DNA probed with labeled *AtMU1* DNA. Genotypes are indicated on top. **C)** Southern blot performed with *HpaII*- (left lanes) and *MspI*-cut (right lanes) genomic DNA that was probed for *MEA-ISR*. Genotypes are indicated on top.

Supplemental References:

Larkin MA, Blackshields G, Brown NP, et al. 2007. Clustal W and Clustal X version 2.0. *Bioinformatics* **23**, 2947-2948.

Supplemental table 1: Primers for expression analysis (Ex) and genotyping (Gt).

locus/primer	Application	5'-Sequence-3'
FDM1	Gt, Ex	CTGAAACACCCTACCGCC
FDM1	Gt, Ex	CCGCCTCATCATCATCATCC
FDM2	Gt, Ex	GAGATACAAGCAGCGCGGC
FDM2	Gt, Ex	CCTTGAGAGTGGCTTTTCTTCC
FDM3	Gt, Ex	CGTGCTCTTGCCAAATACC
FDM3	Gt, Ex	GCACCACAAAGCCAATCC
FDM4	Gt, Ex	GCAATGTATTCCAGAAGAGAGC
FDM4	Gt, Ex	TCTTTCCTCACCACTACGTC
FDM5	Gt, Ex	GTGTGGCTTTTCTTCCTTGC
FDM5	Gt, Ex	CGGCTCTCATGGTAAAAGAACG
IDN2	Gt	GTGTTCTCTCTCTCTCCCATC
IDN2	Gt	GCTTAAAATCACAGTGCTTCCC
IDN2	Ex	CCGGACACTGAACAGTGAGAG
IDN2	Ex	CCTTAGCTTCGATCCACTCTC
IDR1	Gt	GACTCAAAGTTGGCAACCTTAG
IDR1	Gt	GTATGGAAGGCGAATGAAGAG
IDR1	Ex	GCCTTTCGGTTCTCCTCATAG
IDR1	Ex	GCGACCAACGATGAGTATCAAG
IDR1	Ex	GATTGTAGAGGAAGACCAGAGG
IDR2	Gt	GAATCACAAAGAGTTGACTATAC
IDR2	Gt	CTTAGCTTCGATCCACTCTCAC
IDR2	Ex	GTGGATCGAAGCTAAGGGATG
IDR2	Ex	CCTTTCAACTGCTCAACCTC
IDR2	Ex	CACGAGAAGCTTGTGCATCC
IDR2	Ex	GGGGCTTTGCCAACTCATTA
IDR3	Gt, Ex	CCAAGAAACGAGAAGTCTCATC
IDR3	Gt, Ex	GGAGAGTAGGCAAAAGCGG
UBQ5	Ex	ACCCCTTGAGGTTGAATCATC
UBQ5	Ex	GTCCTTCTTTCTGGTAAACGT
LBa1	Gt	TGGTTCACGTAGTGGGCCATCG
LBb1	Gt	GCGTGGACCGCTTGCTGCAACT
RBa1	Gt	GCGGCTGAGTGGCTCTTCCAACGTT
RBb1	Gt	GTCGTTTCCCGCCTTCAGTTTAA
SLB1	Gt	GCCTTTTCAGAAATGGATAAATAGCCTTGCTTCC
